# Cytokine Modulation and Antioxidant Defense Restoration by *Jateorhiza macrantha* (Menispermaceae) Leaf Extracts Mitigate CFA‐Induced Hypernociception, Inflammation, and Pyrexia in Rats

**DOI:** 10.1155/bmri/3876955

**Published:** 2026-08-27

**Authors:** Francis Desire Tatsinkou Bomba, Minette Che Gum, Bibiane Aimee Wandji, Erastus Nembu Nembo, Christian Kuete Fofie, Armel Jackson Seukep, Marius Mbiantcha, Telesphore Benoit Nguelefack, Emmanuel Acha Asongalem

**Affiliations:** ^1^ Department of Biomedical Sciences, Faculty of Health Sciences, University of Buea, Buea, Cameroon, ubuea.cm; ^2^ Department of Animal Biology, Faculty of Science, University of Dschang, Dschang, Cameroon, univ-dschang.org

**Keywords:** antihypernociceptive, ethnomedicine, *Jateorhiza macrantha*, neuro-oxidative stress, persistent inflammatory pain, proinflammatory cytokines

## Abstract

*Jateorhiza macrantha* (Menispermaceae) is used in folk medicine in Africa for gastrointestinal and inflammatory disorders. However, its efficacy against inflammatory pain and associated neuro‐oxidative stress remains uncharacterized. This study evaluated the antihypernociceptive, anti‐inflammatory, and antipyretic properties of *J. macrantha* aqueous (AEJM) and methanolic (MEJM) leaf extracts in a rat model of complete Freund′s adjuvant (CFA)–induced persistent inflammation. Chronic inflammatory pain was induced in male Wistar rats by intraplantar injection of CFA (50 *μ*L). The test extracts were administered orally (100–400 mg/kg) for 14 days. Mechanical, cold, and thermal nociception were evaluated using Von Frey filaments, acetone test, and hot‐plate assay, respectively. Paw edema and rectal temperature were monitored to assess anti‐inflammatory and antipyretic effects. Serum levels of TNF‐*α*, IL‐1*β*, and IL‐6 were quantified, whereas oxidative stress markers (MDA, CAT, SOD, and GSH) were measured in brain and spinal cord tissues. Moreover, qualitative phytochemical profiling was performed. AEJM and MEJM significantly (*p* < 0.05–*p* < 0.001) reduced paw edema and fever, increased paw withdrawal thresholds and thermal latencies. These improvements correlated with a significant downregulation of serum TNF‐*α*, IL‐1*β*, and IL‐6. Furthermore, both extracts significantly lowered MDA levels and increased antioxidant defenses (SOD, CAT, and GSH) in the brain and spinal cord. Notably, AEJM restored the locomotor performance compared with diclofenac. Phytochemical analysis showed flavonoids, saponins, steroids and tannins. AEJM and MEJM exert noteworthy anti‐inflammatory, antihypernociceptive, and antipyretic effects mediated through the concurrent suppression of proinflammatory cytokines and reinforcement of central antioxidant systems. These scientifically validate its traditional use.

## 1. Introduction

Inflammatory pain refers to an enhanced sensitivity to noxious stimuli that arises from tissue injury and the ensuing inflammatory response [1]. It constitutes a major clinical problem and underlies numerous debilitating conditions, including rheumatoid arthritis, cardiovascular diseases, and cancer [2, 3]. Inflammation is a complex biological process involving coordinated interactions among immune cells, blood vessels, and a wide array of soluble mediators, particularly proinflammatory cytokines such as tumor necrosis factor‐alpha (TNF‐*α*) and the interleukins IL‐1*β* and IL‐6 [4].

TNF‐*α*, produced predominantly by innate immune cells such as macrophages and natural killer cells, as well as by adaptive immune T lymphocytes, is a key regulator of both acute and chronic inflammatory responses [5]. Upon binding to its receptors TNFR1 and TNFR2 [6], TNF‐*α* initiates the assembly of multiple intracellular signaling complexes (complexes I, IIa, IIb, and IIc), ultimately leading to activation of the nuclear factor kappa B (NF‐*κ*B) pathway [7]. Activation of NF‐*κ*B promotes transcription of a broad range of inflammatory genes, including TNF‐*α* itself, IL‐1*β*, and IL‐6, thereby amplifying and sustaining the inflammatory cascade [8]. These cytokines directly sensitize peripheral nociceptive nerve terminals, reducing neuronal activation thresholds and increasing afferent excitability. This sensitization manifests clinically as pain elicited by normally innocuous stimuli (allodynia) and exaggerated responses to painful stimuli (hyperalgesia) [9, 10].

Beyond their role in nociception, proinflammatory cytokines function as endogenous pyrogens. By reaching the hypothalamus and stimulating prostaglandin E_2_ (PGE_2_) synthesis through activation of the organum vasculosum of the lamina terminalis (OVLT), these mediators elevate the thermoregulatory set point and induce fever, a hallmark systemic feature of chronic inflammation [11]. Thus, persistent inflammatory conditions are frequently accompanied by a triad of pain, swelling, and fever driven by overlapping molecular and cellular pathways.

Current pharmacological management of chronic inflammation and its systemic manifestations relies primarily on nonsteroidal anti‐inflammatory drugs (NSAIDs), disease‐modifying antirheumatic drugs (DMARDs), corticosteroids, and opioids. Although these therapies are often effective, their long‐term use is limited by significant adverse effects, including gastrointestinal ulceration, cardiovascular complications, renal toxicity, tolerance, and dependence. These limitations have intensified the search for alternative therapeutic strategies, particularly those derived from medicinal plants, which may offer improved safety profiles, affordability, and accessibility [12, 13].

Medicinal plants have played a central role in traditional health care systems for thousands of years [14]. The World Health Organization estimates that approximately 80% of the global population relies, at least in part, on traditional medicine for primary health care needs [15]. Among such plants, *Jateorhiza macrantha* (Hook. f.) Excell and Mendonca (Menispermaceae) are a species native to tropical Africa that is traditionally used in the treatment of diarrhea and inflammatory conditions. Previous pharmacological studies have demonstrated that methanolic leaf extracts of *J. macrantha* possess analgesic and anti‐inflammatory activities in models of acute pain and inflammation [16]. However, despite these findings, the effects of *J. macrantha* on persistent inflammatory pain, particularly its potential antihypernociceptive and antipyretic actions, remain insufficiently explored.

In light of the growing need for safer and more effective treatments for chronic inflammatory pain, the present study is aimed at evaluating the antihypernociceptive, anti‐inflammatory, and antipyretic effects of aqueous and methanolic leaf extracts of *J*. *macrantha* using a complete Freund′s adjuvant (CFA)–induced model of persistent inflammation in rats. This model closely mimics key features of chronic inflammatory pain in humans, thereby providing a relevant platform for assessing both peripheral and central mechanisms underlying the pharmacological actions of the plant extracts.

## 2. Materials and Methods

### 2.1. Plant Material Collection and Extraction

The leaves of *J*. *macrantha* were harvested in March 2025 from the Southwest Region of Cameroon. Botanical authentication and identification were conducted at the National Herbarium in Limbe, Cameroon, by Mr. Elvis Ndive, where a voucher specimen (No. 6958) was deposited for future reference. The harvested plant material was thoroughly washed with running tap water, air‐dried at room temperature for a period of 4 weeks, and subsequently comminuted into a fine powder using a mechanical grinder.

For the preparation of the aqueous extract (AEJM), 500 g of the powdered leaves were subjected to maceration in 5 L of distilled water for 24 h at room temperature, with intermittent manual stirring. The resultant mixture was filtered through Whatman No. 4 filter paper. The filtrate was concentrated and dried in a ventilated oven at 40°C, yielding 67 g of dry extract (13.4% *w*/*w*).

For the methanolic extract (MEJM), 500 g of the leaf powder were macerated in 5 L of 99.9% methanol for 72 h at room temperature. After filtration through Whatman No. 4 paper, the solvent was removed under reduced pressure using a rotary evaporator (45°C), resulting in 83 g of crude extract (16.6% *w*/*w*). Both extracts were stored in airtight containers at −4°C until pharmacological evaluation.

### 2.2. Qualitative Phytochemical Screening

Preliminary phytochemical analysis of AEJM and MEJM was conducted to identify the major classes of secondary metabolites according to standard qualitative protocols [17]. The extracts were screened for the presence of alkaloids, flavonoids, tannins, saponins, steroids, phenolic compounds, and cardiac glycosides.

### 2.3. Experimental Animals and Ethical Considerations

Adult male Wistar rats (3 months old, 200–250 g) were obtained from the animal facility of the Faculty of Health Sciences, University of Buea. The exclusive use of male animals in this study was based on prior pharmacological investigations of *J. macrantha* [16] and reflects a common practice in initial pharmacological screening to minimize hormonal variability; the influence of sex as a biological variable warrants investigation in future studies. The animals were housed in standard ventilated cages under a controlled environment (12 h light/dark cycle and ambient temperature) with ad libitum access to a standard pellet diet and water. All experimental procedures were conducted in strict accordance with international guidelines for the care and use of laboratory animals and received formal approval from the Institutional Animal Ethics Committee of the University of Buea (Ref: UB‐IACUC No. 24/2025). Furthermore, all procedures complied with the ARRIVE 2.0 (Animal Research: Reporting of In Vivo Experiments) guidelines to ensure transparent and complete reporting of the in vivo study.

### 2.4. Chemicals and Reagents

CFA, trichloroacetic acid, thiobarbituric acid, naphthylethylenediamine, sulphanilamide, phosphoric acid, and 5,5 ^′^‐dithiobis (2‐nitrobenzoic acid) (DTNB) were sourced from Sigma‐Aldrich (Germany). Diclofenac sodium was purchased from Denk‐Pharma (Germany). Rat‐specific ELISA kits for TNF‐*α*, IL‐6, and IL‐1*β* were obtained from R&D Systems (United States). All other chemicals and solvents used were of analytical grade.

### 2.5. Induction of Persistent Inflammatory Pain

Baseline values for allodynia (mechanical and cold), thermal hyperalgesia, paw diameter, and rectal temperature were established for each animal prior to induction. Persistent inflammatory pain was induced via a single intraplantar (*i.pl*.) injection of 50 *μ*L of CFA into the left hind paw. The injection site was predisinfected with 70% ethanol to prevent secondary infection. Successful induction of inflammation was confirmed on Day 3 post‐CFA by verifying that all injected animals exhibited a significant increase in paw diameter (≥ 2 mm above baseline) and a reduction in paw withdrawal threshold relative to preinjection values, prior to the commencement of treatment.

### 2.6. Experimental Design and Treatment Protocol

Based on a priori criteria, animals were included in the treatment phase only if they successfully developed CFA‐induced inflammation and excluded if they failed to develop paw edema after the CFA injection. Ultimately, all 54 animals met the inclusion thresholds and completed the study, with no animals, experimental units, or data points excluded from the final analysis. Three days following the induction of inflammation with CFA, the rats were randomly allocated into nine experimental groups, each consisting of six animals (*n* = 6). No a priori statistical power calculation was performed to determine sample sizes; instead, group sizes (*n* = 6) were determined based on historical experimental precedent and established protocols for the CFA‐induced model to minimize animal usage while maintaining scientific validity [18] and is consistent with published pharmacological investigations in this model [19]. Group 1 served as the negative control, receiving distilled water (10 mL/kg, *p.o*.), whereas Group 2 acted as the reference group and was treated with diclofenac sodium (5 mg/kg, *p.o*.). Diclofenac sodium at 5 mg/kg was selected as the reference standard as this dose has been widely validated in the CFA‐induced chronic inflammation model in rats [20] and produces reproducible anti‐inflammatory and antinociceptive effects without causing overt toxicity. Groups 3, 4, and 5 were administered the aqueous extract (AEJM) at doses of 100, 200, and 400 mg/kg (*p.o*.), respectively. Similarly, Groups 6, 7, and 8 received the methanolic extract (MEJM) at the same respective doses of 100, 200, and 400 mg/kg (*p.o*.). Group 9 was designated as the naïve group, receiving only the vehicle without prior CFA induction. This naïve group served as a healthy reference to confirm that behavioral baselines were not intrinsically altered by the oral gavage procedure itself and to verify the magnitude of CFA‐induced changes. All pharmacological interventions were administered once daily via oral gavage over a period of 14 consecutive days. To monitor the progression of the treatment, behavioral and physiological parameters were systematically evaluated on Days 1, 4, 7, 10, and 13 posttreatment initiations. In the present study, daily body‐weight monitoring was not systematically recorded during the treatment period. Future studies will include rigorous, daily physical and body‐weight assessments to formally evaluate the subacute safety profile of the extracts.

### 2.7. Behavioral Assessments of Nociception

#### 2.7.1. Mechanical Allodynia (Von Frey Test)

Mechanical sensitivity was evaluated using calibrated Von Frey filaments [21]. Rats were placed in individual plexiglass chambers with a wire mesh floor and allowed to habituate for 15 min. Filaments (0.4–15 g) were applied perpendicularly to the midplantar surface of the inflamed paw. Following the “up‐and‐down” method, the threshold was defined as the minimum force (g) required to elicit a brisk withdrawal or flinching response.

#### 2.7.2. Cold Allodynia (Acetone Test)

Cold hypersensitivity was assessed by applying 100 *μ*L of acetone to the plantar surface of the affected paw using a micropipette [22]. The duration of nocifensive behaviors (paw licking, shaking, or elevation) was monitored for 60 s. Each paw was tested thrice with 3‐min intertrial intervals to determine the mean response duration.

#### 2.7.3. Thermal Hyperalgesia (Hot‐Plate Test)

Thermal nociception was measured using a hot‐plate apparatus (50°C ± 0.5°C) [23]. The latency to exhibit nocifensive responses (paw lifting, licking, or jumping) was recorded in seconds. A safety cut‐off of 10 s was maintained to preclude tissue injury.

### 2.8. Physiological and Functional Assessments

#### 2.8.1. Paw Edema and Antipyretic Evaluation

Inflammation was quantified by measuring the paw diameter using a digital Vernier caliper (Mitutoyo Digimatic, Japan). The percentage inhibition of edema was calculated with the following equation [20]: *%*edema inhibition = 100 × (1 − Vt/Vc), where Vc and Vt represent the mean edema of the control and treated groups, respectively. Additionally, systemic febrile response was monitored using a digital thermometer to record rectal temperatures at designated intervals [24].

#### 2.8.2. Open Field Test for Locomotor Activity

On Day 14, spontaneous locomotor activity was assessed in an open‐field arena (100 × 100 × 50 cm) [25]. Each rat was placed in the center, and the number of squares crossed with all four paws during a 5‐min observation period was recorded. It is acknowledged that the open‐field test captures both locomotor activity and anxiety‐like behavior; therefore, reduced crossing in the vehicle group may reflect a combination of pain‐induced movement avoidance and anxiety, whereas extract‐induced improvements could partially reflect anxiolytic effects in addition to antinociceptive ones.

### 2.9. Sample Collection and Biochemical Assays

#### 2.9.1. Serum and Tissue Processing

On Day 15, animals were euthanized under deep anesthesia (ketamine 55 mg/kg; diazepam 5 mg/kg). Blood was collected via cardiac puncture and centrifuged (3000 rpm, 15 min) to isolate serum for cytokine quantification (TNF‐*α*, IL‐6, and IL‐1*β*) via ELISA [26]. The brain and spinal cord were rapidly harvested, homogenized (15% *w*/*v*) in ice‐cold Tris‐HCl buffer, and centrifuged (10,000 rpm, 20 min, 4°C). The supernatants were aliquoted for oxidative stress analysis.

#### 2.9.2. Oxidative Stress Markers

Lipid peroxidation was quantified by measuring malondialdehyde (MDA) concentrations [27]. The activity of antioxidant enzymes, including superoxide dismutase (SOD) and catalase (CAT), along with reduced glutathione (GSH) levels, was determined using established spectrophotometric protocols [28].

#### 2.9.3. Statistical Analysis

Data are presented as the mean ± standard error of the mean (SEM) for six animals per group (*n* = 6). Prior to parametric analysis, the normal distribution of continuous variables was confirmed using the Shapiro–Wilk test, and homogeneity of variances was verified. Meeting these assumptions justified the subsequent selected statistical tests. Statistical significance was determined using GraphPad Prism (v5.01). Comparisons between multiple groups were performed using one‐way ANOVA followed by Tukey′s post hoc test. For data involving time‐dependent variables, two‐way repeated‐measures ANOVA followed by Bonferroni′s post hoc test was utilized. A *p* value < 0.05 was considered statistically significant.

## 3. Results

### 3.1. Phytochemical Composition

Qualitative phytochemical screening of *J*. *macrantha* leaf extracts revealed a diverse profile of secondary metabolites (Table 1). Both the aqueous (AEJM) and methanolic (MEJM) extracts tested positive for steroids, flavonoids, tannins, saponins, and phenolic compounds. However cardiac glycosides were found only in MEJM. Notably, alkaloids were absent from both preparations. It is acknowledged that the current phytochemical data are qualitative in nature; quantitative characterization (e.g., total flavonoid content and total phenolic content by spectrophotometric methods) would provide a stronger basis for dose‐response interpretation and is recommended for future studies.

**Table 1:** Qualitative phytochemical profile of *Jateorhiza macrantha* leaf extracts. The relative presence of secondary metabolites was recorded as follows: (+) presence and (−) absence.

*J. macrantha* extracts	Saponins	Flavonoids	Steroids	Tannins	Alkaloids	Cardiac glycosides	Phenols
Aqueous	**+**	**+**	**+**	**+**	**−**	**−**	**+**
Methanol	**+**	**+**	**+**	**+**	**−**	**+**	**+**

*Note:* (**+)** Presence and (−) absence of the metabolites.

### 3.2. Antihypernociceptive Effects on Allodynia and Hyperalgesia

**Figure 1:**
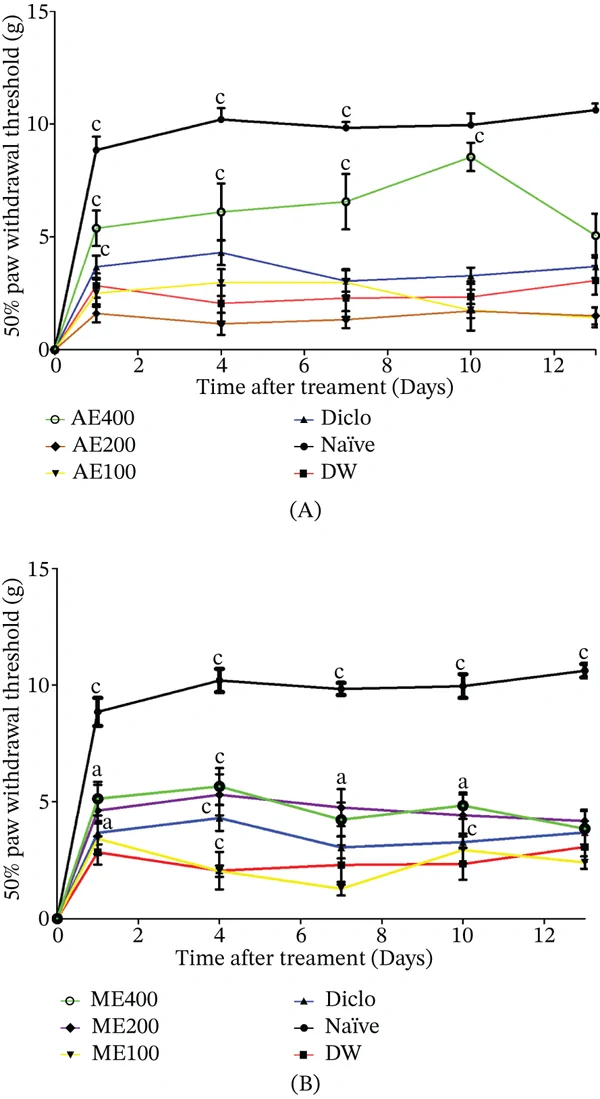
Modulation of mechanical allodynia by *J. macrantha* extracts. Time‐course effects of (A) AEJM and (B) MEJM on paw withdrawal thresholds in the CFA‐induced chronic pain model. Data are expressed as mean ± SEM (*n* = 6). ^a^ *p* < 0.05, ^b^ *p* < 0.01, and ^c^ *p* < 0.001 relative to the negative control (distilled water [DW]). Diclo, diclofenac (5 mg/kg).

**Figure 2:**
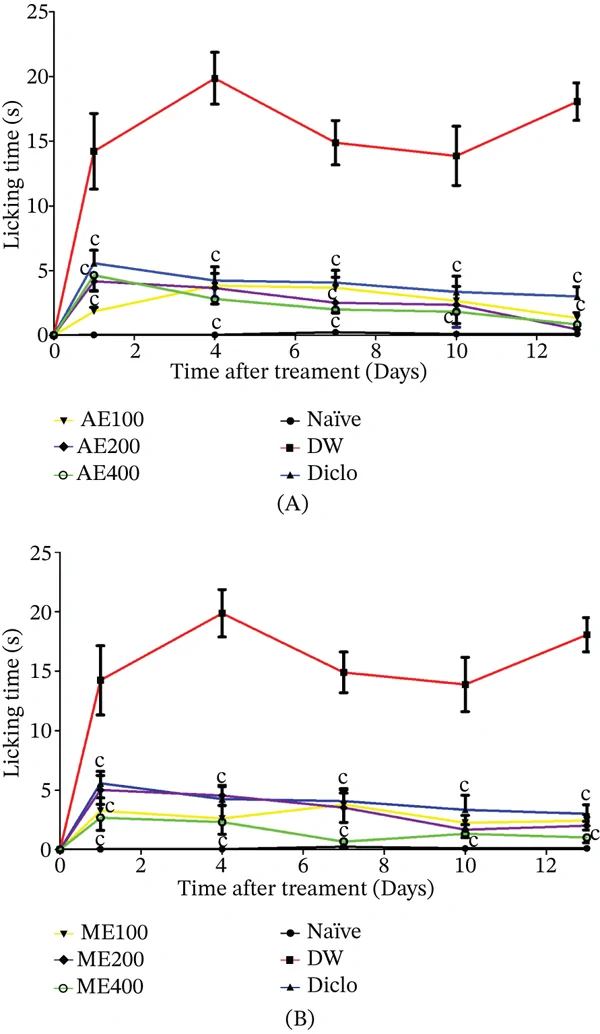
Suppression of cold allodynia by *J. macrantha* extracts. Comparison of the inhibitory effects of (A) AEJM and (B) MEJM on acetone‐induced paw‐licking behavior. Data are presented as mean ± SEM (*n* = 6). Significance levels: ^a^ *p* < 0.05, ^b^ *p* < 0.01, and ^c^ *p* < 0.001 versus control.

**Figure 3:**
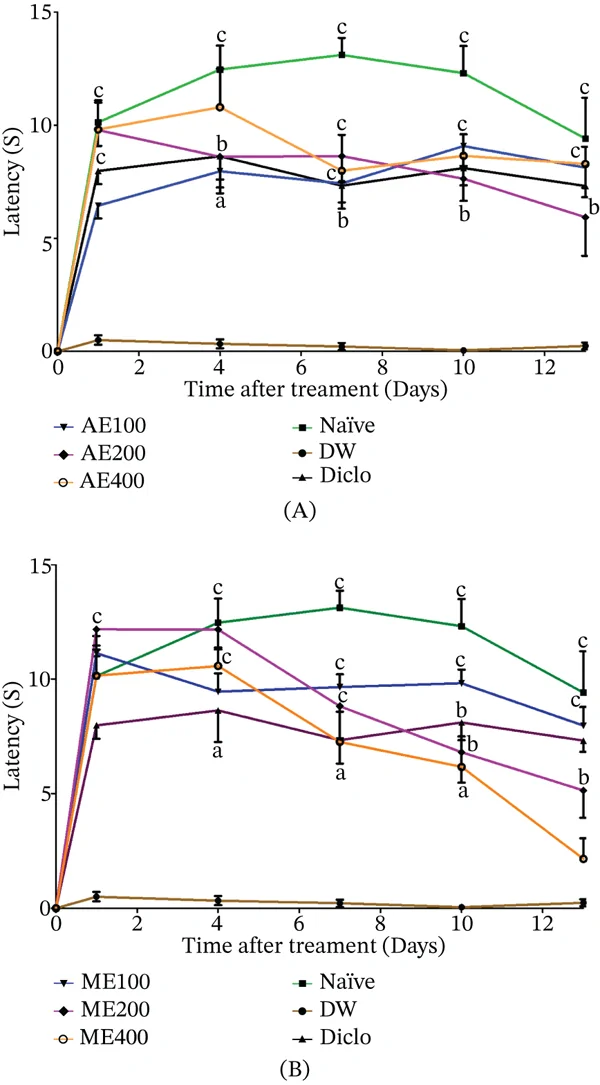
Attenuation of thermal hyperalgesia. Effects of (A) oral AEJM and (B) MEJM on hot‐plate withdrawal latencies. Bars represent mean ± SEM (*n* = 6). ^a^ *p* < 0.05, ^b^ *p* < 0.01, and ^c^ *p* < 0.001 denote statistical significance compared with the vehicle‐treated group.

The administration of CFA successfully induced chronic pain states, which were significantly mitigated by oral treatment with *J. macrantha* extracts in a dose‐ and time‐dependent manner (Figures 1, 2, and 3).

Regarding mechanical allodynia, AEJM (400 mg/kg) exhibited the most robust efficacy, reaching a maximal paw withdrawal threshold increase of 66.6% on Day 10 (*p* < 0.001; Figure 1A). Although MEJM also significantly attenuated mechanical sensitivity (Figure 1B), its peak effects were generally less pronounced than those of the aqueous extract. In comparison, the reference drug diclofenac (5 mg/kg) produced a maximal inhibition of 33.3% on Day 4. It should be noted, however, that diclofenac was employed at a single validated dose (5 mg/kg) in this model, and that a full dose‐response comparison for diclofenac was not performed; accordingly, comparisons between the extracts and diclofenac should be interpreted with caution.

The extracts demonstrated even higher potency against cold allodynia (Figure 2). Repeated administration of both AEJM and MEJM at all doses resulted in a marked reduction in paw‐licking duration (*p* < 0.001). AEJM (200 mg/kg) achieved a near‐complete reversal of cold allodynia with a 97.5% reduction in licking time by Day 13 (Figure 2A), whereas MEJM (400 mg/kg) reached a peak inhibition of 95.5% on Day 7 (Figure 2B). Diclofenac also exhibited significant activity, peaking at 83.4% inhibition.

Thermal hyperalgesia was similarly resolved by both extracts, as indicated by significantly increased withdrawal latencies in the hot‐plate test (*p* < 0.01; Figure 3). The most significant antihyperalgesic response was recorded for AEJM at 200 mg/kg on Day 4 (97.5% inhibition) (Figure 3A), outperforming the peak effect of diclofenac (86.23%) at the dose tested (Figure 3B).

### 3.3. Anti‐Inflammatory and Antipyretic Activities

**Figure 4:**
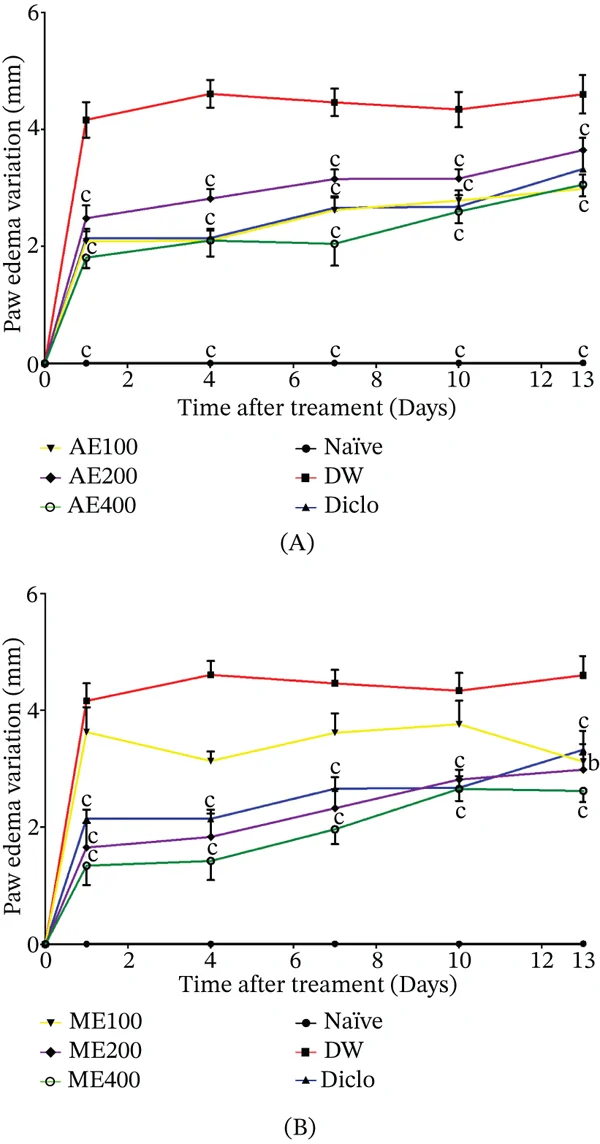
Antiedematous activity in the CFA model. Percentage and absolute variation of paw edema following treatment with (A) AEJM and (B) MEJM. Values are mean ± SEM (*n* = 6). ^a^ *p* < 0.05, ^b^ *p* < 0.01, and ^c^ *p* < 0.001 versus distilled water control.

The anti‐inflammatory potential of *J. macrantha* was evidenced by the significant reduction in CFA‐induced paw edema (Figure 4). MEJM at 400 mg/kg demonstrated the most potent antiedematous effect, achieving a maximal inhibition of 66.4% on Day 4 (*p* < 0.001) (Figure 4B). AEJM also reduced swelling in a dose‐dependent manner (Figure 4A). Both extracts showed greater antiedematous activity than diclofenac (27.5% maximal inhibition); this difference should be interpreted with caution given the use of a single reference dose and the pharmacokinetic differences between crude plant extracts and a purified NSAID.

**Figure 5:**
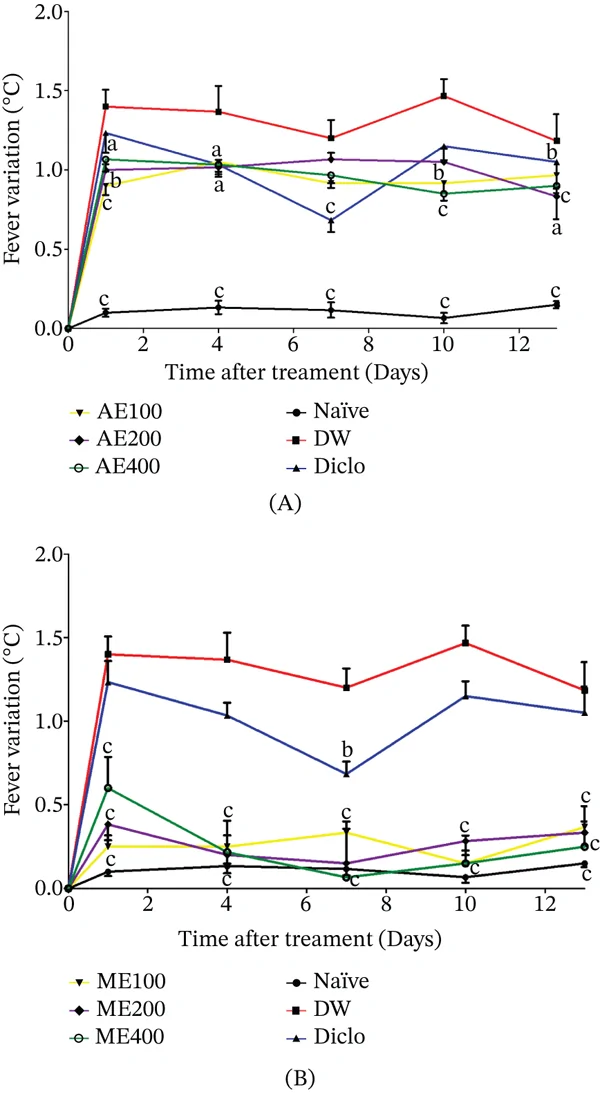
Antipyretic efficacy against CFA‐induced fever. Daily monitoring of rectal temperature changes in rats treated with (A) AEJM and (B) MEJM. Data are represented as mean ± SEM (*n* = 6). ^a^ *p* < 0.05, ^b^ *p* < 0.01, and ^c^ *p* < 0.001 relative to the vehicle group.

Furthermore, AEJM effectively managed the systemic febrile response associated with CFA‐induced inflammation (Figure 5A). Rectal temperatures were significantly lowered compared with the vehicle group (*p* < 0.05–*p* < 0.001), with MEJM (400 mg/kg) producing the most substantial antipyretic effect (93.3% reduction) on Day 7 (Figure 5B).

### 3.4. Modulation of Proinflammatory Cytokines

**Figure 6:**
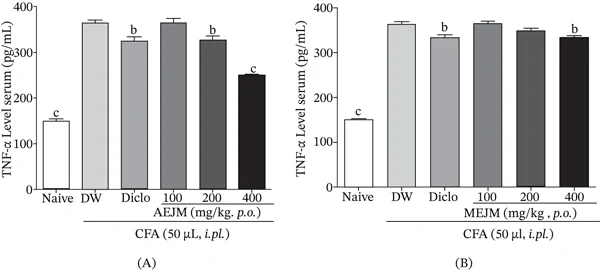
Suppression of serum TNF‐*α* concentrations. Comparative effects of (A) AEJM and (B) MEJM on the levels of the proinflammatory cytokine TNF‐*α*. Each bar denotes mean ± SEM (*n* = 6). Statistical significance: ^a^ *p* < 0.05, ^b^ *p* < 0.01, and ^c^ *p* < 0.001 compared with the control.

The induction of persistent inflammation via CFA resulted in a significant elevation of serum proinflammatory cytokines in vehicle‐treated rats. Chronic oral administration of *J*. *macrantha* aqueous (AEJM) and methanolic (MEJM) extracts significantly attenuated this inflammatory response. Regarding TNF‐*α*, both extracts reduced serum concentrations compared with the vehicle control (*p* < 0.01; *p* < 0.001), with AEJM demonstrating a robust dose‐dependent profile and achieving a peak inhibition of 36.14% at 400 mg/kg (Figure 6). MEJM at 100 and 200 mg/kg did not reach statistical significance for TNF‐*α* suppression (Figure 6B), indicating a less pronounced dose‐response profile for the methanolic extract on this cytokine. The efficacy of AEJM at 400 mg/kg for TNF‐*α* suppression (36.14%) exceeded that of diclofenac (11.8%) (Figure 6A); this difference likely reflects the broader mechanistic profile of the crude extract versus the selective cyclooxygenase (COX)–inhibiting action of diclofenac, rather than an absolute superiority, as direct mechanistic comparisons were not performed.

**Figure 7:**
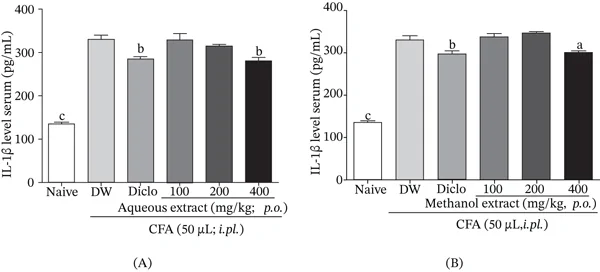
Impact of *J. macrantha* on serum IL‐1*β*. Reduction of serum IL‐1*β* levels by (A) AEJM and (B) MEJM following 14 days of treatment. Bars represent mean ± SEM (*n* = 6). ^a^ *p* < 0.05, ^b^ *p* < 0.01, and ^c^ *p* < 0.001 versus the distilled water group.

**Figure 8:**
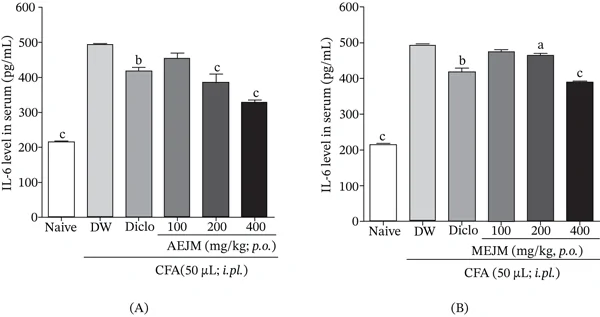
Modulation of serum IL‐6 levels. Effect of (A) AEJM and (B) MEJM on IL‐6 production in CFA‐sensitized rats. Data are represented as mean ± SEM (*n* = 6). ^a^ *p* < 0.05, ^b^ *p* < 0.01, and ^c^ *p* < 0.001 compared with the control group.

Similarly, IL‐1*β* levels were significantly lowered by both AEJM and MEJM at the 400 mg/kg dose (*p* < 0.05 and *p* < 0.01, respectively; Figure 7), with AEJM producing a 15% reduction relative to the control (Figure 7A). Diclofenac also significantly reduced IL‐1*β* (*p* < 0.01; Figure 7B), consistent with the known ability of COX inhibitors to indirectly modulate IL‐1*β* production. Lower doses of both extracts (100 and 200 mg/kg) did not achieve statistically significant reductions in IL‐1*β*. Furthermore, both extracts induced a dose‐dependent decrease in IL‐6 levels (*p* < 0.05–*p* < 0.001), as illustrated in Figure 8. The most potent suppressive effect on IL‐6 was observed in the AEJM 400 mg/kg cohort (33.61%) (Figure 8A), consistently outperforming the methanolic extract across all tested doses. It is noted that MEJM at 100 mg/kg did not reach statistical significance for IL‐6 reduction (Figure 8B).

### 3.5. Mitigation of Oxidative Stress in the Central Nervous System (CNS)

**Figure 9:**
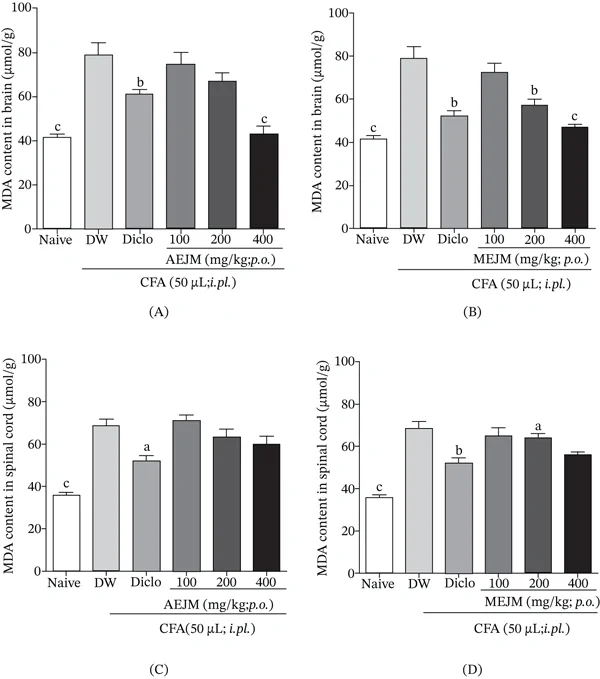
Inhibition of lipid peroxidation in central tissues. Levels of malondialdehyde (MDA) in the (A, B) brain and (C, D) spinal cord following extract administration. Data are presented as mean ± SEM (*n* = 6). ^a^ *p* < 0.05, ^b^ *p* < 0.01, and ^c^ *p* < 0.001 versus negative control.

CFA‐induced systemic inflammation was accompanied by marked oxidative stress within the CNS, characterized by increased lipid peroxidation and the depletion of endogenous antioxidant defenses. As shown in Figure 9, CFA administration significantly elevated MDA levels in both the brain and spinal cord. Repeated treatment with AEJM and MEJM successfully reversed this trend; specifically, MEJM exhibited a superior dose‐dependent reduction in brain tissue, reaching 39.74% inhibition at 400 mg/kg (*p* < 0.001) (Figure 9B), this compares with the aqueous extract (Figure 9A). In the spinal cord, MEJM significantly inhibited lipid peroxidation by 17.64% (*p* < 0.05) (Figure 9D), whereas AEJM did not reach statistical significance in this specific tissue (Figure 9C).

**Figure 10:**
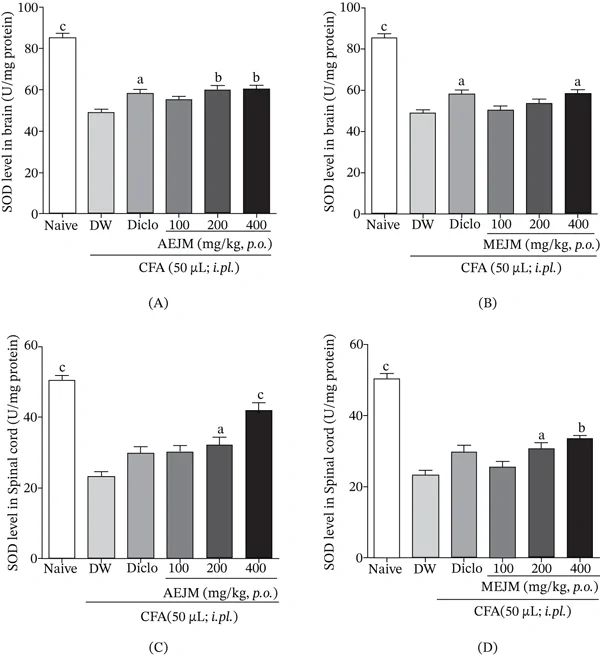
Restoration of superoxide dismutase (SOD) activity. Upregulation of SOD in the (A, B) brain and (C, D) spinal cord by *J. macrantha* leaf extracts. Values represent mean ± SEM (*n* = 6). Significance: ^a^ *p* < 0.05, ^b^ *p* < 0.01, and ^c^ *p* < 0.001 relative to the control.

**Figure 11:**
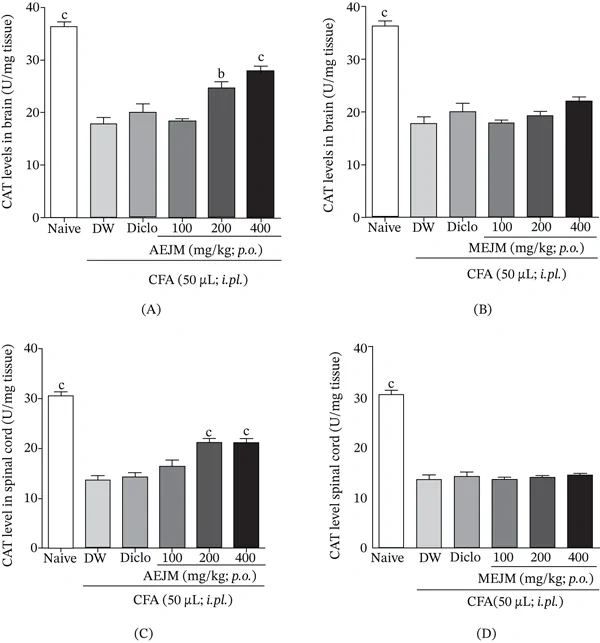
Enhancement of catalase (CAT) activity. Comparative analysis of CAT levels in the (A, B) brain and (C, D) spinal cord of treated rats. Each bar indicates mean ± SEM (*n* = 6). ^a^ *p* < 0.05, ^b^ *p* < 0.01, and ^c^ *p* < 0.001 compared with the vehicle.

The extracts further fortified the antioxidant status of the CNS by upregulating enzymatic and nonenzymatic defenses. Both AEJM and MEJM significantly increased SOD activity in the brain and spinal cord (*p* < 0.05–*p* < 0.001; Figure 10), with AEJM (400 mg/kg) eliciting a remarkable 82% increase in the spinal cord (Figure 10C). In contrast, Diclofenac showed no significant enhancement of SOD in the spinal cord (Figure 10D) and only a marginal 22% increase in the brain (Figure 10A,B). CAT activity was also dose‐dependently enhanced by AEJM (*p* < 0.01), particularly in the brain where a 56.22% increase was observed (Figure 11A). In contrast, diclofenac (5 mg/kg), as expected for a selective COX inhibitor with no direct antioxidant mechanism, did not increase CAT activity in both brain (Figure 11B) and spinal cord (Figure 11C,D).

**Figure 12:**
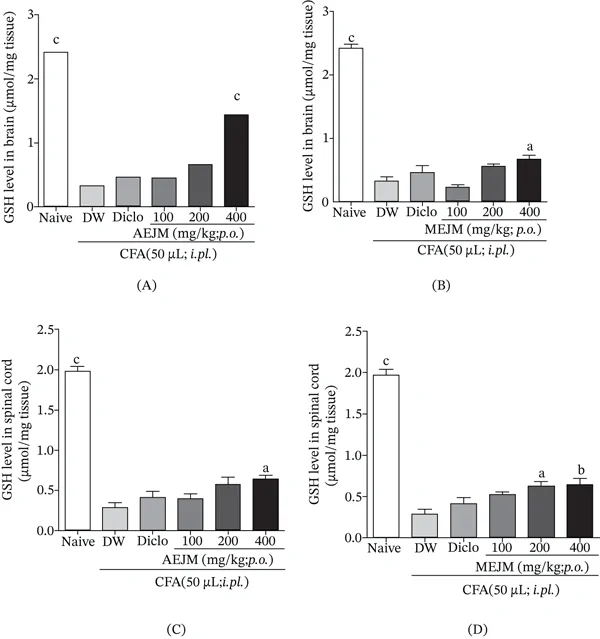
Recovery of reduced glutathione (GSH) stores. Effects of AEJM and MEJM on nonenzymatic antioxidant levels in the (A, B) brain and (C, D) spinal cord. Data are represented as mean ± SEM (*n* = 6). ^a^ *p* < 0.05, ^b^ *p* < 0.01, and ^c^ *p* < 0.001 versus control.

These enzymatic improvements were complemented by significant increases in reduced GSH levels (Figure 12). AEJM at 400 mg/kg produced the most profound restoration of GSH in brain tissue (33.6% increase) (Figure 12A), whereas MEJM exhibited a dose‐dependent effect in the spinal cord, peaking at a 94% increase (Figure 12D). Notably, diclofenac failed to significantly alter GSH levels in either the brain (Figure 12B) or the spinal cord (Figure 12C).

### 3.6. Recovery of Functional Locomotor Activity

**Figure 13:**
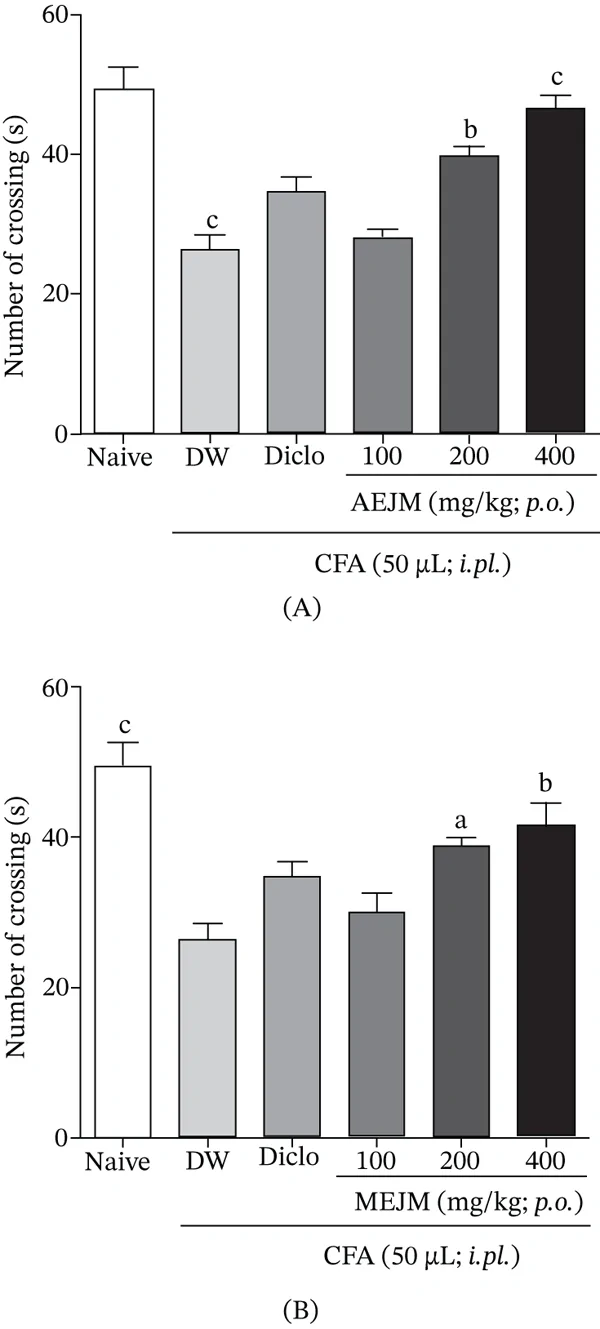
Improvement in functional locomotor performance. Impact of aqueous (A) and methanol (B) extracts on spontaneous movement in an open field arena. Bars reflect the mean ± SEM of six animals. ^a^ *p* < 0.05, ^b^ *p* < 0.01, and ^c^ *p* < 0.001 relative to the CFA‐treated vehicle group.

The state of persistent inflammatory pain in vehicle‐treated rats was characterized by a significant decline in locomotor performance. However, intervention with *J. macrantha* extracts led to a dose‐dependent restoration of mobility (Figure 13). AEJM at 400 mg/kg demonstrated the highest restorative potential, improving locomotor activity by 80% relative to the vehicle group (*p* < 0.001) (Figure 13A). The diclofenac‐treated group did not exhibit a statistically significant improvement in mobility (Figure 13B). This observation, although noteworthy, should be interpreted cautiously; the difference may reflect the broader anti‐inflammatory and antioxidant profile of the crude extracts rather than an intrinsic superiority over NSAIDs. Furthermore, improved locomotion may partly reflect anxiolytic effects of the extracts in addition to pain relief (see Section 2.8.2).

## 4. Discussion

The present study was designed to evaluate the antihypernociceptive, anti‐inflammatory, and antipyretic effects of the aqueous (AEJM) and methanolic (MEJM) leaf extracts of *J*. *macrantha* using a CFA‐induced model of persistent inflammatory pain. *i.pl.* injection of CFA successfully produced a sustained inflammatory state characterized by mechanical allodynia, thermal hyperalgesia, localized edema, systemic fever, and impaired locomotor activity. These clinical features validate the establishment of a chronic inflammatory pain model, which is essential for studying the transition from acute to persistent nociception.

CFA is well recognized for its ability to induce long‐lasting sensitization of primary afferent nociceptors, whose cell bodies are located in the sensory ganglia, particularly the dorsal root ganglia (DRG). This peripheral sensitization is subsequently accompanied by central sensitization within the spinal cord and higher supraspinal structures. These pathological processes are driven largely by the activation of glial cells (microglia and astrocytes), which release a cascade of proinflammatory mediators, including TNF‐*α*, IL‐1*β*, and IL‐6 [19, 29, 30]. The sustained exposure of nociceptive pathways to these mediators lowers neuronal activation thresholds and enhances synaptic transmission efficiency, manifesting as mechanical allodynia and thermal hyperalgesia. Concurrently, these cytokines increase vascular permeability, facilitate leukocyte infiltration, and induce vasodilation, thereby promoting the formation of localized edema [31]. Systemically, proinflammatory cytokines function as endogenous pyrogens that stimulate prostaglandin E_2_ (PGE_2_) synthesis in the hypothalamus via the OVLT. This process elevates the thermoregulatory set point, resulting in the febrile response observed in the vehicle group [12].

In the current study, repeated oral administration of both AEJM and MEJM significantly attenuated CFA‐induced mechanical and cold allodynia, as well as thermal hyperalgesia. These antihypernociceptive effects were accompanied by a marked reduction in paw edema and rectal temperature, indicating that *J. macrantha* possesses robust anti‐inflammatory and antipyretic properties. The behavioral improvements were dose‐dependent and, in several parameters, exceeded those of diclofenac at the tested dose (5 mg/kg). However, since diclofenac was employed at a single clinically validated dose and no dose‐response curve was constructed for the reference drug, and given the fundamental pharmacokinetic differences between a purified NSAID and a crude plant extract, these comparisons should be interpreted as descriptive rather than conclusive.

The phytochemical screening revealed that both extracts are rich in flavonoids, steroids, tannins, saponins, and phenolic compounds. These secondary metabolites are well documented in the literature for their anti‐inflammatory, analgesic, and antioxidant properties [32]. Flavonoids and phenolic compounds are known to interact with COX and lipoxygenase (LOX) pathways, and in certain experimental systems, have been shown to suppress the production of proinflammatory cytokines and modulate intracellular signaling cascades such as NF‐*κ*B. It is important to emphasize that although these mechanistic associations are supported by the broader literature, the present study did not include direct mechanistic assays (e.g., COX activity, NF‐*κ*B western blot, and iNOS expression); the proposed mechanisms therefore remain putative and subject to experimental verification in future studies. Steroidal constituents may further contribute to these effects by stabilizing cellular membranes and limiting the release of inflammatory mediators.

Consistent with these molecular mechanisms, both AEJM and MEJM significantly reduced serum concentrations of TNF‐*α*, IL‐1*β*, and IL‐6. TNF‐*α* is a primary regulator of the inflammatory response, acting through TNFR1 and TNFR2 receptors expressed on immune, glial, and neuronal cells to trigger signaling pathways essential for the development and maintenance of inflammatory pain [7]. IL‐1*β*, synthesized by monocytes, macrophages, and nonimmune cells like endothelial cells, is also expressed within nociceptive DRG neurons [33] and promotes pain hypersensitivity by enhancing PGE_2_ synthesis in both neuronal and glial cells [34]. IL‐6 contributes to the acute‐phase response and acts synergistically with TNF‐*α* and IL‐1*β* to amplify inflammatory signaling and nociceptive sensitization [35]. The coordinated suppression of these cytokines by *J. macrantha* extracts suggests a potential role in dampening both peripheral and central inflammatory processes.

It is notable that the cytokine‐lowering effects of the extracts at the highest doses were generally comparable to or greater than those of diclofenac at the dose tested. This is consistent with the possibility that the phytochemical constituents of *J. macrantha* act through multiple pathways, as opposed to the primarily prostaglandin‐focused mechanism of diclofenac. However, targeted mechanistic studies are needed to confirm this hypothesis. These findings align with previous reports demonstrating the central analgesic activity of *J. macrantha* in the tail‐flick test and its anti‐inflammatory efficacy in carrageenan‐induced models [16].

Chronic inflammatory pain is also inextricably linked to oxidative stress, which both drives and is exacerbated by sustained inflammatory signaling. CFA‐induced inflammation triggers the excessive generation of reactive oxygen and nitrogen species (ROS/RNS), including superoxide anions, hydrogen peroxide, and nitric oxide (NO). This creates a self‐perpetuating cycle of tissue damage and nociceptive sensitization [36, 37]. NO is synthesized by various nitric oxide synthase (NOS) isoforms, neuronal (nNOS), inducible (iNOS), and endothelial (eNOS), with iNOS playing a dominant role in inflammatory conditions following its induction by cytokines and NF‐*κ*B activation [38–40]. Excessive ROS/RNS promote lipid peroxidation, protein oxidation, and DNA damage; MDA serves as a critical marker for such lipid peroxidation and oxidative injury [41]. Thus, proinflammatory cytokines and oxidative stress are undeniably closely interconnected and can influence each other in numerous physiological and pathological processes [42].

In this study, CFA significantly elevated MDA levels in both brain and spinal cord tissues, confirming increased oxidative stress within central pain‐processing regions. Treatment with AEJM and MEJM significantly reduced MDA concentrations, particularly in the brain, suggesting effective attenuation of lipid peroxidation. Parallel to this, both extracts fortified endogenous antioxidant defenses, as evidenced by the increased activity of SOD and CAT, alongside elevated levels of reduced GSH. SOD catalyzes the dismutation of superoxide radicals into hydrogen peroxide, preventing the formation of highly reactive peroxynitrite; CAT then decomposes hydrogen peroxide into water and oxygen, and GSH serves as a vital cellular redox buffer and substrate for GSH peroxidase [43]. The coordinated upregulation of these systems likely contributes to the observed neuroprotective and antihypernociceptive effects.

A noteworthy finding was the differential effect of AEJM and MEJM on spinal cord oxidative stress. Although MEJM significantly reduced MDA in the spinal cord (*p* < 0.05), AEJM did not reach statistical significance in this tissue despite its efficacy in the brain. This discrepancy may be explained by several factors. First, the aqueous and methanolic extracts differ in their phytochemical composition (Table 1); the methanol extract contains steroids and tannins, which may confer greater CNS bioavailability or specific activity in spinal cord tissue. Second, the differential penetration of bioactive constituents across the blood‐spinal cord barrier versus the blood–brain barrier—which have distinct characteristics—could explain the tissue‐specific effects. Third, the spinal cord may represent a pharmacokinetically distinct compartment, requiring different concentrations or physicochemical properties of active molecules for effective antioxidant protection. These hypotheses require further investigation through pharmacokinetic and mechanistic studies.

The antioxidant actions of *J. macrantha* are likely attributable to its rich content of polyphenols and steroids, which are known to scavenge free radicals, chelate metal ions, and modulate the expression of antioxidant enzymes [18, 44, 45] by mitigating oxidative damage; these compounds may further suppress cytokine production and interrupt the reciprocal amplification between oxidative stress and inflammation [42, 46]. Finally, the marked reduction in spontaneous locomotor activity in vehicle‐treated rats reflects both pain‐related movement avoidance and general malaise, and possibly anxiety‐like behavior. The dose‐dependent restoration of locomotor performance by AEJM and MEJM indicates effective pain relief and improved functional status, with a potential contribution from anxiolytic properties that cannot be excluded with the present test battery. The lack of significant locomotor improvement in the diclofenac group at the dose tested is noteworthy, though whether this reflects genuine mechanistic differences or dose‐specific effects cannot be determined without further experimentation.

## 5. Conclusion

The findings of the present investigation demonstrate that the aqueous and methanolic leaf extracts of *J*. *macrantha* exert potent antihypernociceptive, anti‐inflammatory, and antipyretic activities within a CFA‐induced model of persistent inflammatory pain. These therapeutic effects were evidenced by a significant attenuation of both mechanical and thermal hypersensitivity, the successful mitigation of localized paw edema and systemic fever, and the restoration of functional locomotor activity.

At the molecular level, the pharmacological efficacy of these extracts is hypothesized to involve a synergistic modulation of inflammatory and oxidative pathways. This is underscored by the systemic suppression of key proinflammatory cytokines (TNF‐*α*, IL‐1*β*, and IL‐6) and the profound reduction of oxidative stress markers within the CNS. However, although both extracts reduced brain oxidative stress, only MEJM significantly protected the spinal cord against lipid peroxidation.

Collectively, these data provide robust scientific validation for the ethnomedicinal application of *J*. *macrantha* in the management of inflammatory conditions. Furthermore, the results highlight the plant′s potential as a valuable source of bioactive secondary metabolites for the development of novel therapeutic strategies targeting chronic inflammatory pain and neuro‐oxidative injury. Future studies should address the limitations of the current work, including acute and subchronic toxicity assessment, quantitative phytochemical characterization, direct mechanistic investigations (e.g., COX/LOX assays and NF‐*κ*B pathway analysis), histopathological evaluation, pharmacokinetic profiling, and the inclusion of both sexes to fully characterize the pharmacological potential of *J. macrantha*.

## Author Contributions

Francis Desire Tatsinkou Bomba, Minette Che Gum, Emmanuel Acha Asongalem, and Telesphore Benoit Nguelefack: conceptualization, methodology, investigation, data analysis, writing of original draft, and supervision. Minette Che Gum, Bibiane Aimee Wandji, Erastus Nembu Nembo, Christian Kuete Fofie, Marius Mbiantcha, and Armel Jackson Seukep: investigation, writing, review and editing, as well as validation.

## Funding

No funding was received for this manuscript.

## Disclosure

All authors have read and approved the final version of the manuscript.

## Conflicts of Interest

The authors declare no conflicts of interest.

## Data Availability

The data supporting the findings of this study are available from the corresponding author upon request.
